# Low Density of Top Predators (Seabirds and Marine Mammals) in the High Arctic Pack Ice

**DOI:** 10.1155/2016/1982534

**Published:** 2016-09-29

**Authors:** Claude R. Joiris, Karin Boos, Diederik D'Hert, Dominik A. Nachtsheim

**Affiliations:** ^1^Laboratory for Polar Ecology (PolE), 1367 Ramillies, Belgium; ^2^Royal Belgian Institute for Natural Sciences, 1000 Brussels, Belgium; ^3^MARUM-Center for Marine Environmental Sciences, University of Bremen, 28359 Bremen, Germany

## Abstract

The at-sea distribution of top predators, seabirds and marine mammals, was determined in the high Arctic pack ice on board the icebreaker RV* Polarstern* in July to September 2014. In total, 1,620 transect counts were realised, lasting 30 min each. The five most numerous seabird species represented 74% of the total of 15,150 individuals registered: kittiwake* Rissa tridactyla*, fulmar* Fulmarus glacialis*, puffin* Fratercula arctica*, Ross's gull* Rhodostethia rosea*, and little auk* Alle alle*. Eight cetacean species were tallied for a total of 330 individuals, mainly white-beaked dolphin* Lagenorhynchus albirostris* and fin whale* Balaenoptera physalus*. Five pinniped species were represented by a total of 55 individuals and the polar bear* Ursus maritimus* was represented by 12 individuals. Four main geographical zones were identified: from Tromsø to the outer marginal ice zone (OMIZ), the Arctic pack ice (close pack ice, CPI), the end of Lomonosov Ridge off Siberia, and the route off Siberia and northern Norway. Important differences were detected between zones, both in species composition and in individual abundance. Low numbers of species and high proportion of individuals for some of them can be considered to reflect very low biodiversity. Numbers encountered in zones 2 to 4 were very low in comparison with other European Arctic seas. The observed differences showed strong patterns.

## 1. Introduction

The Central Arctic Ocean is one of the least studied regions on earth, mainly due to its inaccessibility caused by permanent ice cover. However, it is already threatened by climatic changes, which calls for a proper assessment and monitoring of this vulnerable ecosystem for future comparisons. There is a strong focus on research in the Arctic marine ecosystem in recent years due to many open questions, for example, regarding the ecology and physiology of polar organisms and the effects of climate change and sea ice decline. In view of these apparent changes, it is essential to monitor the state of an ecosystem to enable comparisons to baseline data and projections into future conditions. However, the distribution of top predators, seabirds and marine mammals, is not well understood, especially in the understudied and permanently ice-covered Central Arctic Ocean. Generally, the at-sea occurrence and density of top predators can be considered to reflect prey availability and thus integrate the structure and functioning of the ecosystems they belong to. In the frame of our long-term study on the distribution of seabirds and marine mammals in polar marine ecosystems, this paper reports on data collected in poorly known areas of the high Arctic pack ice, including Lomonosov Ridge.

## 2. Material and Methods

### 2.1. Counting Method and Environmental Factors

The quantitative at-sea distribution of marine mammals and seabirds was established during the PS86 (AURORA) expedition of icebreaker RV* Polarstern* from Tromsø and back, from 8 July to 3 August 2014, and the PS87 expedition from Tromsø to Bremerhaven, from 5 August to 8 October 2014, respectively. Transect counts lasted half an hour without width limitation from the bridge at 18 m above sea level on a continuous basis, allowing light and visibility; see description and discussion in [1, 2]. The animals were detected with the naked eye, and observations were confirmed and complemented with binoculars when useful. Photographic documents were also used, especially for rare species or those difficult to identify in the field.

Water temperature (sea surface temperature, SST), salinity, and fluorescence (chlorophyll) were continuously recorded on board at subsurface sampling (keel, −10 m). Ice cover was evaluated from the bridge and expressed as % coverage within an approximated range of 500 m around the ship.

Basic data have been included in the biodiversity datasets: http://ipt.biodiversity.aq/archive.do?r=rbins_joiris_2014_ps86_birds_mammals and http://ipt.biodiversity.aq/archive.do?r=rbins_joiris_2014_ps87_birds_mammals, respectively.

### 2.2. Statistical Analysis

We tested the effects of the different zones on the abundance of selected species using a generalized linear mixed model (GLMM with Poisson-distributed error terms and log-link function) into which the fixed effects factors “zone” and “species” were included. The species were selected according to their total abundances and chosen, where numbers exceeded 200 individuals. This selection was important in order to warrant stable convergence of the model. Because the data originated from two different cruises, we included “cruise” as a random effects factor to account for grouped data per cruise. We tested and confirmed overdispersion by including an additive dispersion term as a random factor to the GLMM, which comprised as many factor levels as observations [4]. The variance of the (random) additive dispersion term was considerably higher than zero indicating overdispersion in the data. Also, the Bayesian Information Criterion (BIC) revealed the model including the additive dispersion term to be clearly more parsimonious than the original model (BIC_add_ = 12956.71; BIC_orig_ = 46410.72). For this reason, we applied a negative binomial mixed model in order to account for overdispersion. Although there were many zeros in excess, we ruled out zero-inflation on the basis that the zero-inflation value was, generally by a factor of at least 6, smaller than the model's estimated coefficients and, therefore, negligibly small (using the argument “zeroInflation = T”; zero-inflation = 1.0*∗*10^−6^; and SE = 4.064*∗*10^−9^). We refrained from including the interaction term into the model, because data replicates were not available for all combinations of zones and species. Hence, the inclusion of an interaction term would lead to severe overparametrisation and possibly false results. We therefore focused solely on the two main effects.

The final model was checked for stability through comparing the empirical data with 1000 randomly generated simulations of negative binomial distributions based on the present model predictions. The empirical data lay perfectly within the range of the simulated data. Therefore, the model was considered robust. Significance of the main effects was established by applying likelihood ratio tests (LRT), where the deviance of the respective full models was compared with that of the corresponding reduced models not comprising the respective factor of interest using the R function “anova.” All models were fitted in R, version 3.2.2 [5], using the function “glmer” from the R package “lme4” [6] and the function “glmmadmb” from the R package “glmmADMB” [7, 8].

A separate analysis was conducted to test for the repeatability of the findings of the qualitative and quantitative species composition along the three consecutive subzones 1a, 1b, and 2a. Just as described above, we selected the species according to their overall abundance (i.e., >200 individuals). This led to the exclusion of Ross's gull from the selected species. For the same reasons as above, we chose a negative binomial model to account for overdispersion and ruled out zero-inflation as described above (zero-inflation = 1.0*∗*10^−6^; SE = 5.0*∗*10^−9^). Instead of including the random factor “cruise,” we included the “revisit” of each subzone as a random factor to account for grouping in the data. Model stability check was conducted as described above and showed a clear deviation from running within the expected ranges and, thereby, far more optimistic predictions than provided by the empirical data. Because the model did not converge properly with both factors included, we, therefore, refrained from keeping the factor “species” in the model and pooled the data for the three subzones. The comparisons between species are presented in a descriptive way.

## 3. Results

**Figure 1:**
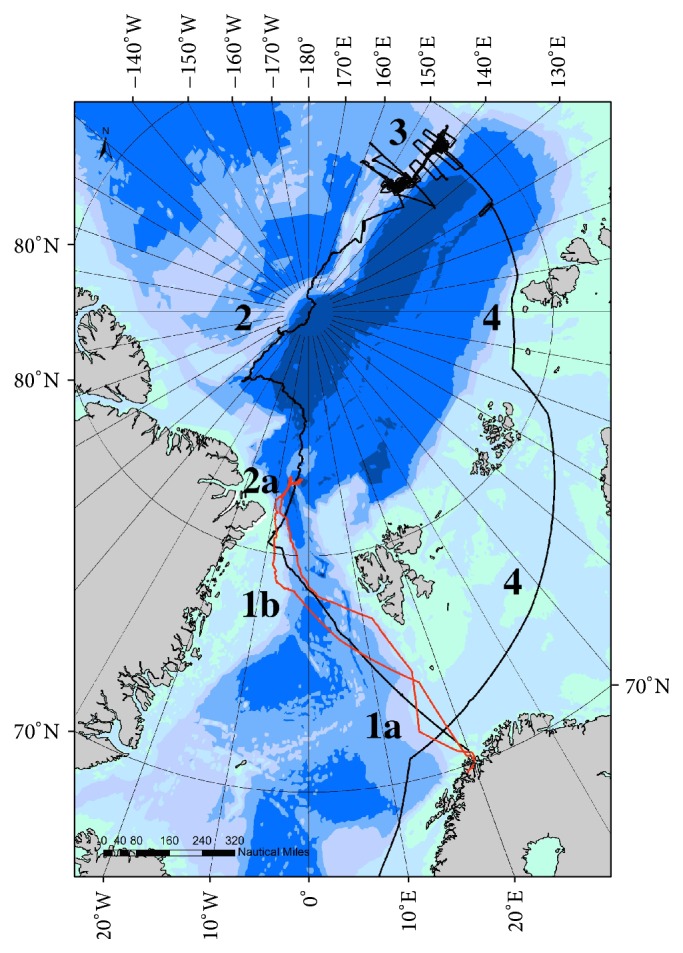
Route of the PS86 (red) and PS87 (black) expeditions of RV* Polarstern* in July–September 2014; for definition of the zones, see text.

During the 1,620 transect counts devoted to top predator census on board the RV* Polarstern*, expeditions PS86 and PS87 (*partim* North of 66°30′N) (Figure 1), a total of 15,150 birds were detected, belonging to 23 species, that is, an average of 9.3 identified individuals per count (Table 1). Five species represented the vast majority: kittiwake* Rissa tridactyla*, fulmar* Fulmarus glacialis*, puffin* Fratercula arctica*, Ross's gull* Rhodostethia rosea*, and little auk* Alle alle* with 2.2, 1.5, 1.3, 1.1, and 0.7 birds per count, respectively, representing 74% of the total. They were followed by ivory gull* Pagophila eburnea* and Brünnich's guillemot* Uria lomvia* (0.3 and 0.2 per count). Eight cetacean species were counted for a total of 330 individuals, that is, 0.2 per count. The most numerous species were white-beaked dolphin* Lagenorhynchus albirostris* (0.15 per count) and fin whale* Balaenoptera physalus* (0.02 per count). Five pinniped species were represented by 55 individuals (0.03 per count), of which 0.01 were harp seals* Pagophilus groenlandicus* and 0.01 hooded seals* Cystophora cristata*. Polar bear* Ursus maritimus* was recorded in low numbers (12 individuals including a mother with two large cubs and another one with three small cubs plus one out of effort).

**Figure 2:**
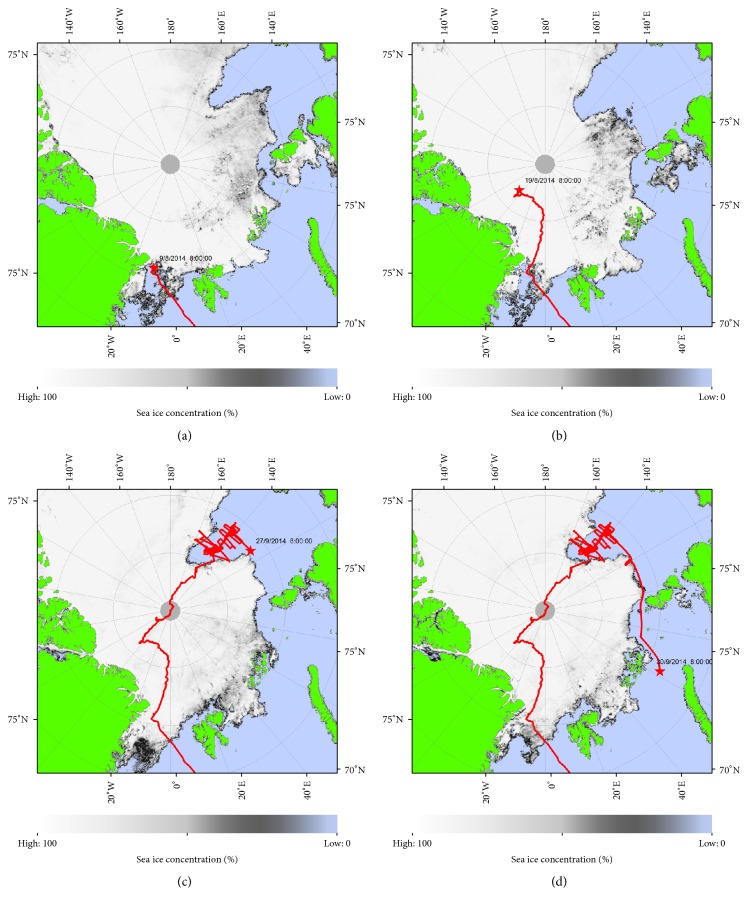
Ice conditions during the PS86 and PS87 expeditions of RV* Polarstern* in July–September 2014; ship's position indicated by a star: 9 August (a); 19 August (b); 27 September (c); and 30 September (d). Institute of Environmental Physics, University of Bremen, Germany: https://seaice.uni-bremen.de/amsr2/index.html.

Four major geographical zones were defined, mainly on the basis of ice coverage: zone 1 from Tromsø to the outer marginal ice zone (OMIZ), passing through the North-East Water (NEW) polynya. Zone 2 covered the ice-covered close pack ice (CPI), including the Lomonosov Ridge separating the Amerasian and Eurasian Basins. Zone 3 was basically the ice-free part of the Lomonosov Ridge off the New Siberian Islands and zone 4 the coastal waters off Siberia to northern Norway (Figure 1). Ice conditions in the four zones are illustrated in Figure 2. Characterisation of the four zones and observations of the most numerous (main) species are shown in Table 2. Geographical differences were obvious. The analysis included the eight most numerous species (each >200 individuals). It revealed both factors “zone” and “species” to be highly significant and, therefore, having an effect on the observed abundances. Independent of the individual species, the results showed that the highest number of selected top predators was found in zone 1 (mean numbers per count, all eight species pooled: 7.92), followed by zone 4 (5.89), and the least amounts were found in zones 3 and 2 (3.38 and 1.17, resp.) (LRT_zone_: deviance = 608.66, *df* = 13, and *p* < 0.0001).

**Figure 3:**
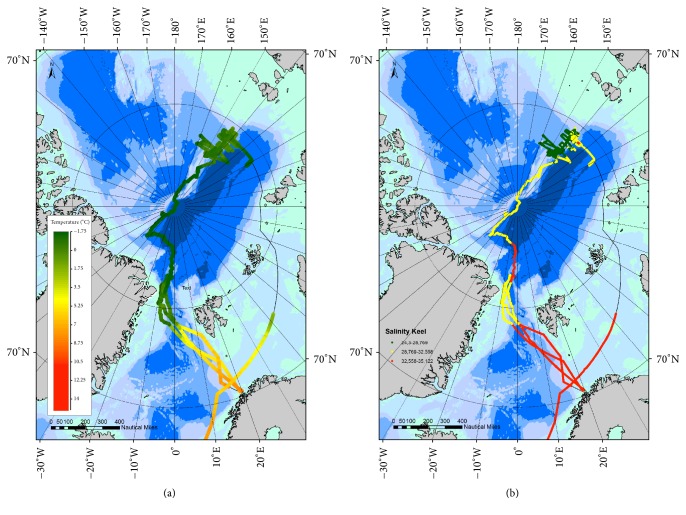
Environmental conditions registered during the PS86 and PS87 expeditions of RV* Polarstern* in July–September 2014: water temperature (SST, °C) (a); salinity (b).

These important differences seem to reflect differences in biological productivity, considering chlorophyll to be a good index: maximal values were 12.7, 4.1, 2.3, and 3.9, respectively, in the four zones (Table 2, Figure 3).

Pooled over all zones, kittiwake presented the highest numbers (2.12 per count), including three individuals belonging to the Siberian subspecies* R. t. pollicaris* (zone 3, 81.1°N). It was followed by fulmar (1.46) and little auk (0.66). Considerably lower but similar numbers were found for ivory gull and puffin (0.33 and 0.31, resp.). Lowest numbers were detected for Brünnich's guillemot (0.33), Ross's gull (0.14), and white-beaked dolphin (0.15) (Table 2). Overall, puffins and white-beaked dolphins were almost exclusively found in zone 1a (*n* = 506 and 239, resp.). The pattern described above for the overall abundances in the four zones was true for Brünnich's Guillemots, fulmars, little auks, puffins, and white-beaked dolphins. This pattern was reversed in kittiwakes and ivory gulls, which were sighted in highest numbers in zone 4. Ross's gulls were the only species found in highest numbers in zone 3. The main species in zone 1 were fulmar (4 individuals per count, mean value), little auk (2 per count), kittiwake (0.5) and Brünnich's guillemot (0.45), white-beaked dolphin (0.4), and fin whale (0.07). Using chlorophyll (fluorescence) as an indicator, biological productivity is also the highest in this area. The ice-covered area (zone 2) showed by far the lowest densities, with significant numbers of kittiwakes (0.2) and ivory gulls (0.04), as well as polar bears (0.02). Biological productivity (chlorophyll) was low. Zone 3 showed low numbers as well: Ross's gull (1.2) and ivory gull and kittiwake (1 each) being the most abundant; biological productivity (chlorophyll) was low as well. Zone 4 showed intermediate abundances: highest numbers of kittiwakes (5 per count, including higher concentrations at the local ice edge around 130°E; see Figure 2(c)), ivory gulls (0.3), and fulmar (0.2); harp seals were present at a few stations only (0.03, 120°E).

In addition to the above, a “first” zone was covered three times during both expeditions and divided into three subzones (subzones 1a, 1b, and 2a; see Figure 1), deserving special attention. Clear geographical differences were detected (Table 3). Pooled together, top predators were highest in zone 1a (12.6 per count), followed by zones 1b (7.6) and 2a (2.1). In zone 1a (Greenland Sea), fulmar was the most numerous (7.55 per count) followed by puffin (2), Brünnich's guillemot (0.9), kittiwake (0.7), white-beaked dolphin (0.9), and fin whale (0.1), with high productivity (chlorophyll) of 2.8. In zone 1b (NEW polynya), species were present with little auk (6.4), fulmar (1.2), and ivory gull (0.7), with productivity of 0.9. In zone 2a (CPI), the present species were ivory gull (1), fulmar (0.8), kittiwake (0.2), hooded seal (0.1), and polar bear (0.03) with intermediate productivity (1.2). The tested reproducibility of data between successive transects was high for some species, reflecting good reproducibility of the counting method, fulmar, Brünnich's guillemot, ivory gull, and kittiwake, but not for other species, reflecting the heterogeneity of their distribution, little auk and puffin. The analysis of the selected and pooled species revealed the factor “subzone” to be significantly effective (LRT_subzone_: deviance = 8.52, *df* = 5, and *p* = 0.014). In fact, the results showed that subzones 1a and 1b were significantly similar with respect to the abundances (*p* = 0.13), even though the abundances in subzone 1a were higher than those in subzone 1b. This is most likely due to high variations and many excess zeros in the data. Subzone 2a hosted clearly less individuals than the subzones 1a and 1b (*p* = 0.0001 and 0.013, resp.).

## 4. Discussion

Not only were mammal numbers very low but they were moreover very limited spatially: dolphins and fin whales in zone 1a, especially along the shelf slope, where autumn aggregations were already encountered [10]. Hooded seal and polar bear were present on the OMIZ, zone 2a, and harp seal in a few counts off Siberia (zone 4, OMIZ, 120°E).

Two of the three high Arctic gulls deserve special comments, Sabine's gull* Xema sabini* being absent in our counts, one observation only, out of effort.

The breeding range of the ivory gull includes the Canadian Arctic, Greenland, Svalbard, and Russian Arctic islands [11]. Northeast (NE) Greenland in particular seems to be a hotspot for breeding sites [12], apparently due to the vicinity to an attractive feeding ground: the NEW polynya. It usually breeds in colonies, either inland on steep cliffs and nunataks or coastal on barren islands and lowlands. In rare cases, it uses gravel-covered sea ice close to the coast as breeding platform: a breeding site on an ice floe covered with gravel was discovered in Independence Fjord, NE Greenland [13]. We recently reported an even more extreme breeding habitat: a gravel-covered iceberg 70 km off NE Greenland, close to the NEW polynya (81°N, 9°W) [14]. During this study, 60 more adults plus an unknown number of chicks, not included in our calculations, were detected (the position is indicated by a star on Figure 2(a)). Dramatic population declines have already been observed in Canada and Greenland during the last decades [12, 15–17]. Our own data, collected from the same observation platform with the same methodology, show a decrease in ivory gull abundance in the Greenland Sea from a mean of 1.7 individuals per 30 min count in the 1990–1993 period [9] to 0.4 and 0.3 in 2008 and 2011, respectively [10, 18, 19]. Its relative abundance deeply changed as well; it was one of the three most abundant species in the 1990s, together with fulmar and kittiwake, but now does not often even belong to the top ten any more. The ivory gull is listed as “Endangered” under the Species at Risk Act in Canada and as “Near Threatened” by the International Union for Conservation of Nature (IUCN) Red List of Threatened Species [20, 21]. The global population is estimated to be between 19,000 and 27,000 individuals with still high uncertainties due to its occurrence in highly remote areas [21]. Its breeding range includes the Canadian Arctic, Greenland, Svalbard, and Russian Arctic islands. Northeast (NE) Greenland in particular seems to be a hotspot for breeding sites [11, 12, 22–24], possibly due to the vicinity to an attractive feeding ground: the NEW polynya. In this frame the numbers encountered in zones 3 (0.7 per count) and 4 (0.3 per count) represent a significant part of the world population, probably belonging to the Russian/Siberian breeding sites. Half of them were juveniles and 10% immature: this seems to reflect a good breeding success for this population.

**Figure 4:**
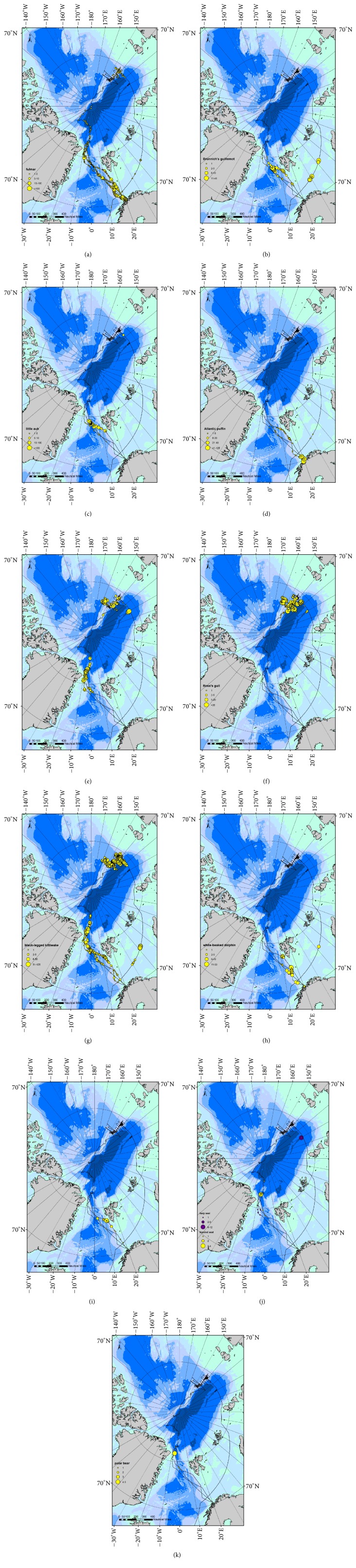
Distribution maps of the main top predator species encountered during the PS86 and PS87 expeditions of RV* Polarstern* in July–September 2014: fulmar* Fulmarus glacialis* (a), Brünnich's guillemot* Uria lomvia* (b), little auk* Alle alle* (c), puffin* Fratercula arctica* (d), ivory gull* Pagophila eburnea* (e), Ross's gull* Rhodostethia rosea* (f), kittiwake* Rissa tridactyla* (g), white-beaked dolphin* Lagenorhynchus albirostris* (h), fin whale* Balaenoptera physalus* (i), harp seal* Pagophilus groenlandicus* and hooded seal* Cystophora cristata* (j), and polar bear* Ursus maritimus* (k).

Ross's gulls are breeding mainly in the deltas of the Kolyma and Khroma rivers, Siberia (142° to 160°E). The world population is estimated at 45,000 to 55,000 individuals, probably >27,000 and possibly 100,000 [22–24]. Adults used to be regularly observed in the Greenland Sea at the end of the breeding season during the 1990s (end of June; Table 4; [9]) but not after 1994 (this team: numerous papers and unpublished data). It is not clear how far this had to be interpreted as movements following breeding failure or as large nonbreeding population. In this study, they represent the most abundant species in zone 3, off its main Siberian breeding grounds, with a total of 190 individuals (1.2 per count). The majority were breeding adults (60%), 25% juveniles, and some nonbreeding adults close to the end of the breeding season in late July-August [22–25]. This again seems to reflect a good breeding success for this species. More information can be found in Figure 4.

**Figure 5:**
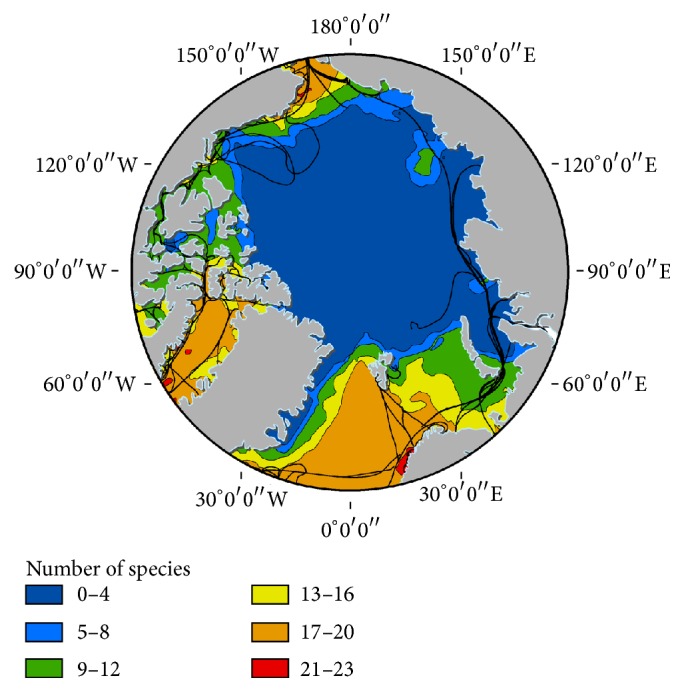
Seabird species diversity of 27 modelled seabird species in the Arctic with shipping lanes overlain: Figure  2(a) in [3], with authorization.

A comparison between our seabird data and the model predictions of Arctic seabird distribution [26] shows a full compatibility in trends: modelled bird diversity varied from 17–20 species in our zone 1 (more than 20 close to the coast) to 0–4 in the high Arctic pack ice (our zones 2 and 4), with a slight increase corresponding to our zone 3 (Figure 5, [3]). Our values were however much lower, mean numbers varying from 1.85 species per count in zone 1 (6 maximum) to 1.95 in subzone 1a, followed by 0.68 in zone 3 (3 maximum), 0.23 in zone 4 (5 maximum), and 0.14 in zone 2 (3 maximum); in all zones, many counts were free of contact. This quantitative discrepancy can partially be explained by the limited period and area covered by our study, while the model was based on broader datasets concerning more species.

## 5. Conclusion

Two factors can be considered the reflection of very low biodiversity: the very low numbers of species and the high relative numbers of individuals for some of them. This was especially the case for zones 2 (Arctic pack ice CPI), 3 (Siberian end of the Lomonosov Ridge), and 4. Numbers of species and individuals were high in zone 1: Greenland Sea and Fram Strait for fulmar, little auk, puffin, and Brünnich's guillemot, as well as white-beaked dolphin and fin whale, comparable to the values usually obtained in the area (e.g., [9, 10, 18]). Ivory gull was present in all zones, with maxima being in zones 2 and 4. Kittiwake was present in all zones as well, with low abundance in zone 2 (CPI) and very high abundance in zone 4. The main conclusion is that numbers were very low for all species in zones 2 to 4, with the exception of Ross's gull in zone 3 (Tables 2 and 3). The observed geographical differences seem to reflect differences in biological productivity of the ecosystems, as illustrated by differences in chlorophyll (fluorescence) data. These baseline data could be integrated in other datasets [27] and in broader discussions, for example, about changing ice conditions and human activities.

These findings provide confirmation of the profound difference between both polar regions. The Arctic pack ice is generally characterised by low top predator density, while Antarctic pack ice is supporting high numbers of penguins, mainly Adélie* Pygoscelis adeliae *and chinstrap* P. antarctica*, and of seals, the majority being crabeater seals* Lobodon carcinophaga *scattered on CPI [28]. Such a major difference might be due to differences in sea ice conditions and as a consequence in ecological structure, the Arctic being mainly covered by multiyear ice and the Antarctic by one-year ice. This situation is in full evolution as a consequence of climate changes. In the Arctic, summer pack ice is strongly reduced, while winter pack ice decreases in a very limited extent only. As a result, the Arctic might be changing into a one-year pack ice ecosystem. The possible ecological consequences of this situation are difficult to evaluate for the time being.

## Figures and Tables

**Table 1:** Seabirds and marine mammals encountered during the PS86 and PS87 expeditions of RV *Polarstern* in 2014; total numbers recorded; mean per count^a^; and *n* = number of 30 min counts.

Species	*Species*	Expedition>	PS86		PS87^b^		All	
		Period>	July		Aug-Sept		July–Sept	
		*n* >	387		1235		1622	
			Sum	Mean	Sum	Mean	Sum	Mean
Fulmar light	*Fulmarus glacialis*		920	2.31	362	0.29	1282	0.79
Fulmar dark	*Fulmarus glacialis*		835	2.16	323	0.26	1158	0.71
Fulmar all	*Fulmarus glacialis*		1755	4.53	721	0.43	2476	1.53
Manx shearwater	*Puffinus puffinus*		1				1	
Fork-tailed storm-petrel	*Oceanodroma furcata*				2		2	
Common guillemot	*Uria aalge*		14		1		15	
Brünnich's guillemot	*Uria lomvia*		93	0.23	262	0.18	355	0.22
Guillemot unidentified	*Uria sp. (aalge/lomvia)*		4		18		22	
Little auk	*Alle alle*		498	1.25	568	0.46	1066	0.66
Black guillemot	*Cepphus grylle*		9		76	0.05	85	
Puffin	*Fratercula arctica*		273	0.69	1829	1.48	2102	1.30
Razorbill	*Alca torda*				51	0.04	51	
Gannet	*Sula bassana*		2		4		6	
Great skua	*Stercorarius skua*		3		4		7	
Pomarine skua	*Stercorarius pomarinus*		2		7		9	
Arctic skua	*Stercorarius parasiticus*		8		25		33	
Long-tailed skua	*Stercorarius longicaudus*		31				31	
Ivory gull	*Pagophila eburnea*		200	0.50	332	0.27	532	0.33
Glaucous gull	*Larus hyperboreus*		11		18		29	
Herring gull	*Larus argentatus*		5		27		32	
Great black-backed gull	*Larus marinus*		14		17		31	
Lesser black-backed gull	*Larus fuscus*		2		5		7	
Kittiwake	*Rissa tridactyla*		278	0.68	3338	2.70	3616	2.23
Sabine's gull	*Xema sabini*				1^c^			
Ross's gull	*Rhodostethia rosea*				1770	1.22	1770	1.09
Arctic tern	*Sterna paradisaea*		11		2		13	
Tern unidentified	*Sterna sp. (hirundo/paradisaea)*				15		15	
Phalarope unidentified	*Phalaropus sp.*				1		1	
∑*birds * ** (**identified)			**3214**	9.19	**11932**	7.17	**15146**	9.34

Blue whale	*Balaenoptera musculus*		6				6	
Fin whale	*Balaenoptera physalus*		34	0.085	4		38	0.02
Minke whale	*Balaenoptera acutorostrata*				1		1	
Humpback whale	*Megaptera novaeangliae*		7		1		8	
Sperm whale	*Physeter macrocephalus*		9		2		11	0.01
Killer whale	*Orca orca*		9				9	
Large whale unidentified	*Cetacea*		35		6		41	0.03
Narwhal	*Monodon monoceros*				7		7	
White-beaked dolphin	*Lagenorhynchus albirostris*		215	0.540	34	0.03	249	0.15
Dolphin unidentified	*Delphinidae*		3		14		17	0.01
∑*cetaceans* (identified)			**280**	0.723	**49**	0.03	**329**	0.20

Bearded seal	*Erignathus barbatus*		5				5	
Harp seal	*Pagophilus groenlandicus*		5		18	0.01	23	0.01
Ringed seal	*Pusa hispida*		7		2		9	
Hooded seal	*Cystophora cristata*		16	0.038	2		18	0.01
Walrus	*Odobenus rosmarus*				1^c^			
Seal unidentified	*Pinnipedia*		36		18		54	0.03
∑*pinnipeds* (identified)			**33**	0.085	**22**	0.01	**55**	0.03

Polar bear	*Ursus maritimus*		4 + 1^c^		8 + 1^c^		9	
Polar bear tracks	*Ursus maritimus*		1		64	0.07	65	0.04
Arctic fox/wolf tracks	*Canidae*				3		3	

^a^For mean values above 0.2 per count for birds and above 0.01 for mammals; ^b^partim North of 66°30′N; ^c^out of effort.

**Table 2:** Data obtained in the four main geographical zones defined during the PS86 and PS87 expeditions of RV *Polarstern*, July–September 2014; *n*: number of 30 min counts; total number; mean per count^*∗*^; and mean *N* per km (see text and Figure 1).

Zone	*n*	Salinity	Temperature °C	Ice cover %	Fluorescence (chlorophyll)	Speed knots	Fulmar		Brünnich's guillemot		Little auk		Puffin		Ivory gull	
							*Fulmarus glacialis*		*Uria lomvia*		*Alle alle*		*Fratercula arctica*		*Pagophila eburnea*	
		Mean (min–max)	Mean (min–max)	Mean (min–max)	Mean (min–max)	Mean (min–max)										
							Total	Mean	Total	Mean	Total	Mean	Total	Mean	Total	Mean
1	419	33.4 (29.3–35.1)	3.3 (−1.7–12.1)	10 (0–80)	2.1 (0.06–12.7)	9.2 (2.8–12.7)	2121	5.06	252	0.6	1054	2.52	509	1.21	78	0.57
2	449	31.1 (29.2–33.3)	−1.4 (−1.7–1.3)	75 (0–100)	1.2 (0.1–5.5)	4.4 (1.1–11.2)	116	0.26	1		4		0		156	0.35
3	160	26.2 (24.3–29.9)	0.2 (−1.3–1.11)	80 (0–100)	1.1 (0.7–2.3)	4.9 (1.2–10.5)	0		1		0		0		105	0.97
4	580	28.6 (24.3–35.0)	0.9 (−1.7–10.8)	2 (0–10)	1.3 (0.6–3.9)	5.7 (2.3–12.2)	126	0.22	100	0.17	8		2		162	0.28

**Zone**	**Ross's gull**			**Kittiwake**		**White-beaked dolphin**		**Fin whale**		**Harp seal**		**Hooded seal**		**Polar bear**		
	*Rhodostethia rosea*			*Rissa tridactyla*		*Lagenorhynchus * *albirostris*		*Balaenoptera physalus*		*Pagophilus groenlandicus*		*Cystophora cristata*		*Ursus maritimus*		
	**Total**		**Mean**	**Total**	**Mean**	**Total**	**Mean**	**Total**	**Mean**	**Total**	**Mean**	**Total**	**Mean**	**Total**		**Mean**

1	0			239	0.57	239	0.57	37	0.09	4	0.01	2	0.01	3		0.01
2	0			112	0.25	0		0		2		14		5		0.01
3	190		1.19	155	0.97	0		0		0		0		0		
4	38		0.07	2931	5.05	10	0.02	1		17	0.03	0		0		

^*∗*^For mean values above 0.1 per count for birds and above 0.01 for mammals.

**Table 3:** Observations of top predators during the same transect visited three times, between Tromsø and the high Arctic pack ice (83°N): main species; total number; mean per count^*∗*^; and mean *N* per km.

Zone^*∗∗*^	*Polarstern *expedition		*n*	Latitude °N	Longitude °E	Salinity	Temperature °C	Ice cover %	Fluorescence (chlorophyll)	Speed knots	Fulmar			Brünnich's guillemot			Little auk		
											*Fulmarus glacialis*			*Uria lomvia*			*Alle alle*		
						Mean (min–max)	Mean (min–max)	Mean (min–max)	Mean (min–max)	Mean (min–max)	Total	Mean	N/km	Total	Mean	N/km	Total	Mean	N/km
1a	PS86		101	70.1/78.4	20.2/−2.4	34.7 (33.2–35.1)	7.1 (2.5–10.6)	0		11.2 (6.0–11.5)	407	4.0	0.36	21	0.21	0.02	4		
1a	PS86		89	70.7/77.8	6.0/19.0	34.3 (34.2–35.1)	8.6 (2.7–12.0)	0		10.9 (3.9–12.7)	1140	12.8	1.17	62	0.75	0.07	460	4.30	0.39
1a	PS87		68	70.2/77.98	20.2/2.44	34.6 (32.5–35.1)	8.0 (3.2–12.4)	0		9.0 (4.1–11.0)	424	5.7	6.19	150	2.21	0.24	32	0.47	0.05
∑1a			258	70.1/78.4	20.2/−2.4	34.5 (32.5–35.1)	7.9 (2.5–12.4)	0	2.79 (0.08–12.7)	10.4 (3.9–11.5)	1971	7.64	0.76	233	0.90	0.09	496	1.92	0.18
1b	PS86		28	78.5/79.2	−2.80/−7.3	30.25 (29.9–31.7)	−0.63 (−1.3–0.31)			6.3 (3.8–9.0)	31	1.1	0.17	0			10	0.08	0.1
1b	PS86		53	79.2/81.3	6.0/−5.6	31.15 (29.6–35.1)	0.39 (−0.97–3.73)			8.9 (4.9–11.5)	73	1.38	0.16	2			28	0.39	0.04
1b	PS87		83	78.1/81.5	2.17/−6.65	30.22 (29.3–33.8)	0.34 (−1.1–4.3)			5.99 (2.80–7.50)	89	1.07	0.18	11	0.13	0.02	528	6.36	1.06
∑1b			164	78.1/81.5	2.17/−6.65	30.54 (29.3–35.1)	0.03 (−1.3–4.3)	17 (0–80)	0.88 (0.47–2.3)	7.06 (2.80–11.5)	193	1.18	0.16	13	0.08	0.01	566	3.45	0.49
2a	PS86		115	79.2/82.9	−5.7/−9.8	30.54 (29.9–31.5)	−0.86 (−1.30–1.60)			6.1 (3.8–9.0)	94	0.82	0.13	1			4		
2a	PS87		32	81.5/82.9	−4.52/−6.61	31.06 (29.3–31.1)	−1.47 (−0.97– −1.70)			4.4 (2.50–6.10)	19	0.59	0.13	0			0		
∑2a			147	79.2/82.9	−9.8/−4.52	30.80 (29.30–31.5)	−1.16 (−0.97– −1.70)	70 (20–90)	1.19 (0.13–5.5)	8.4 (2.50–9.0)	113	0.77	0.09	1			4		

**Zone** ^*∗∗*^	**Puffin**			**Ivory gull**			**Kittiwake**			**Wh.-beaked dolphin**			**Fin whale**			**Harp seal**			**Hooded seal**
	*Fratercula arctica*			*Pagophila eburnea*			*Rissa tridactyla*			*Lagenorhynchus* *albirostris*			*Balaenoptera physalus*			*Pagophilus groenlandicus*			*Cystophora cristata*
	**Total**	**Mean**	**N/km**	**Total**	**Mean**	**N/km**	**Total**	**Mean**	**N/km**	**Total**	**Mean**	**N/km**	**Total**	**Mean**	**N/km**	**Total**	**Mean**	**N/km**	**Total**

1a	157	1.6	0.14	1			49	0.50	0.04	8			4			0			0
1a	116	1.3	0.12	0			117	1.3	0.12	207	2.3		30	0.3	0.03	0			0
1a	1824	26.8	2.98	0			16	0.24	0.03	24			3	0.05	0.005	0			0
∑1a	2097	8.13	0.78	1			182	0.71	0.07	239	0.94		37	0.14	0.01	0			0
1b	0			8	0.28	0.04	5	0.18	0.03				0			3	0.11	0.01	0
1b	0			14	0.26	0.02	64	1.21	0.14				0			0			1
1b	3			55	0.66	0.11	134	1.61	0.27				0			1			2
∑1b	3			77	0.47	0.07	203	1.18	0.16				0			4			3
2a	0			143	1.24	0.20	33	0.29	0.05				0			2	0.02	0.003	14
2a	0			4			23	0.72	0.16				0			0			0
∑2a	0			147	1.00	0.12	56	0.38	0.045				0			2			14

^*∗*^For mean values above 0.1 per count for birds and above 0.01 for mammals.

^*∗∗*^From south to north: 1a, open water; 1b, NEW polynya; and 2a, pack ice (OMIZ: outer marginal ice zone).

**Table 4:** Observations of Ross's gulls *Rhodostethia rosea* in the NEW polynya area; *n* = number of 30 min counts [9].

Date		Year	*n*	Ross's gull	
From	To			Total	Mean/count
25 May	2 June	1993	239	0	
9 June	19 June	1991	451	0	
28 June	31 July	1993	529	92	0.18
21 July	5 August	1993	113	107	0.95
22 July	10 August	1992	25	20	0.80
